# Temporomandibular involvement in children and adolescents with juvenile idiopathic arthritis: a 2-year prospective cohort study

**DOI:** 10.1038/s41598-024-56174-3

**Published:** 2024-03-06

**Authors:** Malin Collin, Nikolaos Christidis, Stefan Hagelberg, Linda Z. Arvidsson, Tore A. Larheim, Malin Ernberg, Britt Hedenberg-Magnusson

**Affiliations:** 1 Division of Oral Diagnostics and Rehabilitation, Department of Dental Medicine, Karolinska Institutet, 141 04 Huddinge, Sweden; 2 Department of Orofacial Pain and Jaw Function, Folktandvården, Sörmland AB, Mälarsjukhuset, 611 32 Nyköping, Sweden; 3 Department of Women’s and Children’s Health, Karolinska Institute, Karolinska University Hospital, 17176 Stockholm, Sweden; 4 Department of Maxillofacial Radiology, Institute of Clinical Dentistry, University of Oslo, Oslo, Norway; 5 Department of Orofacial Pain and Jaw Function, Folktandvården Stockholms Län AB, 11382 Stockholm, Sweden

**Keywords:** Dentistry, Health services, Paediatrics, Prognosis

## Abstract

This study aimed to clinically evaluate temporomandibular joint (TMJ) involvement in juvenile idiopathic arthritis (JIA) and the ability to identify and/or predict development of TMJ-deformities over time using cone beam computed tomography (CBCT). The predictive value of self-reported TMJ pain was also assessed. A prospective longitudinal cohort study comprising 54 children with JIA, 39 girls and 15 boys, was performed. All children had active disease at baseline, 50% with the subtype oligoarthritis. Repeated clinical orofacial and CBCT examinations were performed over a two-year period. At baseline, 39% had radiographic TMJ deformities (24% unilateral, 15% bilateral), at 2-year follow-up, 42% (*p* > 0.05). Both progressing and improving TMJ deformities were observed. An association was found between TMJ-deformities and self-reported TMJ pain at baseline (*p* = 0.01). Maximum unassisted mouth opening (MUO) was smaller for children with TMJ-deformities (*p* < 0.05). The prevalence of palpatory muscle pain was high (48–59%) but not predictive of development of TMJ-deformities. TMJ noises increased over time and crepitations were associated with TMJ-deformities (*p* < 0.05). In conclusion, in children with JIA, self-reported TMJ pain and dysfunction were common and predictive of TMJ deformities. TMJ deformities were associated with smaller MUO and palpatory TMJ pain as well as crepitations. *Trial registration*. ClinicalTrials.gov Protocol id: 2010/2089-31/2.

Juvenile idiopathic arthritis (JIA) is the most common rheumatic disease in childhood. The overall incidence has been reported to be 0.8–23/100,000^1^, in Nordic countries, the incidence is about 15/100,000^2^.

In JIA, the temporomandibular joint (TMJ) is frequently affected^3–5^. TMJ involvement causes pain and functional limitations but can appear with discrete signs and symptoms, i.e., it can go unnoticed^3,4,6–8^. Over time, TMJ arthritis can cause growth disturbances, evoke pain, compromise masticatory function, and affect quality of life in both the short- and long-term perspective^9–13^. To avoid these complications, clinicians should strive for early identification and treatment of TMJ arthritis. In clinical practice, however, it is challenging to diagnose JIA-induced TMJ arthritis and TMJ deformities (arthritis-related alteration of the anatomy of the TMJ) in younger children. Symptoms can be few or atypical and there is an overlap in clinical findings with those of temporomandibular disorders (TMD)^14^. Furthermore, in children, variations in normal anatomy and growth must also be considered.

The introduction of biologic disease modifying antirheumatic drugs (DMARDs) for the management of JIA has led to a great improvement in disease outcome^15,16^. However, recent prospective longitudinal studies have shown that self-reported orofacial pain and functional disability^17^ as well as dentofacial deformities are still common^13,17^ and that TMJ involvement according to magnetic resonance imaging (MRI) is highly prevalent in JIA children both with and without symptoms^18^.

There is a need for comprehensive longitudinal prospective studies to understand the impact of current pharmacological treatment on TMJ involvement. In addition, validated examination methods are needed. Recommendations for what to include in the clinical orofacial examination of children with JIA have been published recently^19,20^. These include clinician-assessed pain location, TMJ pain on palpation, mandibular deviation at maximal mouth opening, maximal unassisted mouth opening capacity, frontal facial symmetry, and facial profile. Longitudinal assessment of mouth opening capacity has also been recommended for evaluation of disease activity^20,21^. Apart from clinical variables, TMJ imaging with cone beam computed tomography (CBCT), which is considered the gold standard for assessing bony TMJ components^22^, and MRI, which is the only imaging modality that reliably can identify TMJ arthritis^23–25^, are often used to assess TMJ status. However, to determine validated cutoff values for clinical variables as well as validated predictors for identifying individuals at high risk of TMJ involvement in JIA are warrant.

Therefore, this study has two aims: to evaluate a set of clinical variables and their ability to identify and/or predict development of TMJ involvement using CBCT as outcome variable and also to investigate the predictive value of self-reported TMJ pain for presence and for development of TMJ deformities.

## Results

### Participants

At baseline, the referring pediatric rheumatologists judged that 39 girls and 15 boys had active disease, 50% with the subtype oligoarthritis. The mean number of years with diagnosis was 4.1 (3.5). Descriptive data from baseline for participating children are presented in Table 1 and data on pharmacological treatment over the study period are presented in Table 2.

**Table 1:** Participant characteristics from baseline, n = 54. Data are presented as mean ± SD or number of children.

Age (years)	All	10.7 ± 2.1
	Girls	10.8 ± 2.1
	Boys	10.6 ± 2.3
Duration of JIA (years)	All	4.1 ± 3.5
	Girls	4.2 ± 3.7
	Boys	3.9 ± 3.2
JIA subtype	Oligoarthritis	25
	Polyarthritis RF neg	18
	Polyarthritis RF pos	1
	Enthesitis-related arthritis	1
	Psoriatic arthritis	5
	Systemic arthritis	3
	Undifferentiated	1

*JIA* juvenile idiopathic arthritis; *RF* Rheumatoid factor.

**Table 2:** Type of pharmacological treatment the children received over the two-year study period, n = 54.

Medication n (%)	Baseline	1-year follow-up	2-year follow-up	*p*
NSAID	23 (42.6)	20 (37.0)	13 (24.1)	**0.023**
sDMARDs	22 (40.7)	22 (40.7)	18 (33.3)	0.183
Methotrexate	22 (40.7)	21 (38.9)	16 (29.6)	0.209
Salazopyrine	–	1 (1.9)	–	0.368
Plaquenil	–	–	2 (3.7)	0.135
bDMARDs	11 (20.4)	14 (25.9)	20 (37)	0.001*
Cortisone	4 (7.4)	4 (7.4)	3 (5.6)	0.895
No medication	12 (22.2)	12 (22.2)	12 22.2)	0.939

*sDMARDs* synthetic disease-modifying antirheumatic drugs; *bDMARDs* biological disease-modifying antirheumatic drugs: Adalimumab (Anti-TNF alfa), Etanercept (Anti TNF-alfa), Anakinra (Anti-IL1 beta), Abatacept (Anti CD-28), Rituximab (Anti-CD20).

Exact Cochran’s Q-test (*p* < 0.05), post hoc testing with multiple McNemar’s exact with Bonferroni correction (*p* < 0.0167).

Significant values in bold.

*Significant difference between baseline and the 2-years follow-up (*p* = 0.002).

### Self-reported pain

At all three examinations, participants reported joint pain, mean ± SD number of painful joints being 2.4 ± 1.9, 1.9 (2.3), and 1.7 (2.1), respectively. The most frequently reported painful joints were the knees (37%), followed by the ankles (25%), and the TMJ (9%). Among those reporting joint pain, the median (IQR) pain intensity was 3 (6.0). The number/frequency of children reporting no joint pain increased significantly, from nine to 20 individuals (*p* = 0.004), at the two-year follow-up; the most significant change taking place between baseline and one-year follow-up.

Self-reported TMJ pain and dysfunction before baseline were reported by 24 (44%) children. A reduction in self-reported TMJ pain and/or dysfunction was observed, from 21 children at the baseline examination to 13 at both the one-year and the two-year follow-up. However, this reduction was not significant (*p* > 0.05). Further analyses with Fisher’s exact test showed a significant association between TMJ deformity and self-reported TMJ pain at baseline (*p* = 0.01). A multinominal logistic regression showed no predictive value of self-reported previous TMJ pain/dysfunction at baseline for developing TMJ changes.

### Imaging findings

**Figure 1:**
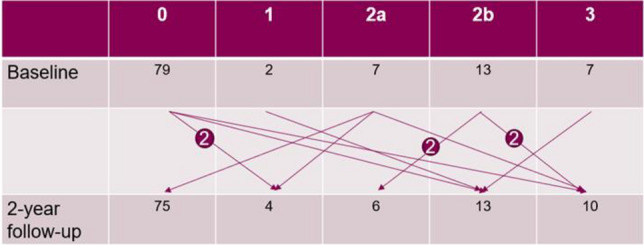
Change in grading of temporomandibular joint deformity (0, 1, 2a, 2b, 3) evaluated with cone beam computed tomography. Data presented on the joint level, an arrow representing one joint unless specified with a number.

At baseline, 61% of the children had no TMJ deformity, whereas 24% showed unilateral and 15% bilateral TMJ deformities. Over time, no significant changes occurred in the grading of TMJ deformity on the group level (*p* > 0.05). On the joint level, five joints received a lower grade, while eight joints received a higher grade of deformity at the two-year follow-up examination (Fig. 1).

An ordinal logistic regression did not show any association between a change in maximum unassisted mouth opening (MUO) without pain and a change in the grade of TMJ deformity over the two-year study period–OR 1.036 (95% CI, 0.925 to 1.159), Wald χ2(1) = 0.368, *p* = 0.543. There was no significant difference in the proportion of TMJ deterioration and no TMJ deterioration in relation to a reduction in MUO without pain (Fischer’s exact test, *p* = 1.00). A multinominal regression showed that it was more likely that children with a larger number of involved joints at baseline would develop TMJ deformity (*p* = 0.04).

Binominal logistic regression showed that an increased duration with disease (*p* = 0.023) as well as smaller MUO without pain (*p* = 0.008) were associated with an increased likelihood of exhibiting TMJ deformity.

### Clinical findings

Mandibular range of motion is presented in Table 3. The children had a mixed dentition, but all had their permanent upper incisors in place. At baseline, 52% of the children had an Angle class I occlusion and 48% had an Angle class II. Over time, the number of children with Angle class I increased and reached 60% at the two-year follow-up. No correlation was found between Angle class II and TMJ deformity, neither at baseline (*p* < 0.586), nor at the two-year follow-up (*p* = 1.000). Midline deviations were found in 28% of the children, but there was no correlation between midline deviation and TMJ deformity, either unilateral or bilateral (*p* = 0.406). There was, however, a significant increase in the frequency of midline deviation over time, from 28% at baseline to 48% at the two-year follow up (*p* < 0.009).

**Table 3:** Change in range of motion from baseline to the two-year follow-up divided by presence of TMJ deformities on CBCT at baseline (n = 54).

N = 54	Baseline Mean (SD)	Year 2 Mean (SD)	*p*
TMJ deformities			
MUO without pain	38.2 (7.2)	43.3 (6.8)	**0.017**
MUO with pain	43.3 (5.2)	46.7 (6.0)	**0.003**
LTR right	8.9 (2.6)	9.4 (1.6)	0.201
LTR left	8.8 (2.0)	9.5 (1.7)	0.088
PTR	7.2 (1.4)	8.1 (2.0)	0.067
No TMJ deformities			
MUO without pain	44.6 (8.7)	44.6 (9.0)	0.644
MUO with pain	50.1 (5.3)	52.0 (6. 0)	**0.003**
LTR right	10.0 (2.0)	10.7 (1.6)	**0.019**
LTR left	10.2 (1.7)	9.8 (1.8)	0.200
PTR	9.0 (1.7)	9.3 (2.1)	0.776

*TMJ* temporomandibular joint; *CBCT* cone beam computed tomography; *MUO* maximum unassisted mouth opening; *LTR* laterotrusion; *PTR* protrusion.

Significance level *p* < 0.05, Wilcoxon Signed rank test.

Significant values in bold.

On a group level, a slight increase in overall mandibular range of motion occurred over the two-year study period. The median (IQR) MUO with pain increased from 47.5 (7.0) mm at baseline to 50.0 (10.0) mm at two-year follow-up (*p* < 0.001). In addition, laterotrusion to the right was significantly increased (*p* < 0.005) at the two-year follow-up (Friedman test and Bonferroni post hoc test). An increase was also observed for laterotrusion to the left, but post hoc testing showed no significant difference between the years. No other significant changes in mandibular range of motion were found.

When the children were clustered by presence of TMJ deformity on CBCT, there was still a significant increase in MUO with pain for both groups between baseline and the two-year follow-up. In addition, laterotrusion to the right significantly increased in the group without TMJ deformity. No other significant changes in mandibular range of motion were found (Table 3).

When MUO with and without pain was analyzed for differences between children with TMJ deformity and those without, we found significant differences between the groups both at baseline and at the two-year follow-up (Table 4).

**Table 4:** Differences in MUO between children with and without TMJ deformity on CBCT at baseline and at two-year follow-up.

	No TMJ deformity Median (IQR)	TMJ deformity Median (IQR)	*p*
Baseline			
MUO without pain	46.0 (10.0)	39.0 (12.0)	**0.003**
MUO with pain	49.0 (6.0)	43.0 (8.0)	**0.000**
2-year follow-up			
MUO without pain	45.5 (9.0)	42.5 (10.0)	**0.008**
MUO with pain	52.0 (9.0)	46.5 (8.0)	**0.001**

*MUO* maximum unassisted mouth opening; *TMJ* temporomandibular joint; *CBCT* cone beam computed tomography. Significance level *p* < 0.05, Mann–Whitney U.

Significant values in bold.

In addition, we found a weak positive association between the calculated difference in MUO (MUO with pain–MUO without pain) and TMJ deformity as seen on CBCT at two-year follow-up examination (*p* = 0.036). However, this association was not found at the baseline examination (*p* = 0.964).

Palpatory TMJ pain was present in 59% of the children at baseline, in 54% at the one-year follow-up, and in 37% at the two-year follow up. The change over time was not significant (*p* > 0.05). When combining palpatory and TMJ deformity findings, the odds ratio for having palpatory TMJ pain was 43% higher (OR 1.425, CI 0.538, 3.774) for children with TMJ deformity on CBCT than for children without TMJ deformity on CBCT. However, the area under the ROC curve for crepitation as a measure of finding TMJ deformities was 0.563 (95% CI, 0.473 to 0.653).

We found that TMJ noises increased over time. On the joint level, crepitations increased significantly from 3.7% to 14.8% over the two-year study period (*p* = 0.045/post hoc test *p* = 0.004). In addition, a corresponding increase in TMJ clickings occurred from 6.4% to 13.9% although this increase was not significant (*p* = 0.013/post hoc test *p* = 0.031, significance level: 0.016). Crepitation was associated with TMJ deformity on CBCT (*p* = 0.005).

The frequency of myalgia in the masseter muscle was 59% at baseline and 48% at the two-year follow-up. For the temporalis muscle the frequency was 22% at both baseline and the two-year follow-up. Our data did not show any significant change over time in palpatory pain in the masseter or the temporalis muscles (*p* > 0.05). Furthermore, a binominal logistic regression could not ascertain any effect of palpatory muscle pain (masseter, temporalis, or pterygoideus lateralis) on the likelihood of finding TMJ deformity on CBCT (*p* > 0.05).

### Additional visits and treatments between the follow-ups

Apart from the scheduled study examinations during the two-year study period, 23 (42%) (year 1) and 15 (28%) (year 2) of the participants received additional examinations and treatments at the Department of Orofacial Pain and Jaw Function at Eastmaninstitutet, Folktandvården Stockholm AB. However, only two of these visits were due to ongoing TMJ arthritis. The remaining visits were due to participants needing treatment for TMD or bruxism or requiring a consultation with a pediatric rheumatologist or an orthodontist.

During the study period, 15 joints were diagnosed with arthritis (during a disease relapse with multiple joints affected or confirmed with MRI or ultrasound) and subsequently treated with local corticosteroid injections (methylprednisolone 40 mg/ml with lidocaine 10 mg/mL, Pfizer, Sollentuna, Sweden). Of these injections, two were administrated by the responsible caregiver at the Department of Orofacial Pain and Jaw Function at Eastmaninstitutet, Folktandvården Stockholm AB and 13 by a pediatric rheumatologist at ALB Astrid Lindgren Children’s Hospital, Karolinska University Hospital, Solna.

## Discussion

This prospective two-year longitudinal cohort study set out to investigate what variables can identify and predict JIA-related TMJ involvement identified as TMJ deformity on CBCT. Our findings showed that there are differences in mandibular range of motion between children with JIA with and without TMJ deformity and that a smaller MUO without pain at baseline was predictive of finding TMJ deformity on CBCT. Crepitations and palpatory TMJ pain were also associated with TMJ deformity. Furthermore, we showed that self-reported TMJ pain and dysfunction were common and that a higher proportion of TMJ deformity was found in children self-reporting TMJ pain.

The previous notion that TMJ arthritis and TMJ involvement in JIA are silent conditions has already been challenged^26^. Similarly, our results show that TMJ pain and dysfunction are common in JIA. Self-reported TMJ pain could in this case be interpreted as functional pain due to TMJ deformities or TMJ arthritis, but it could also be associated with TMD. Previous studies have shown that children with JIA have higher prevalence of TMD compared to healthy children^6,27^. Our data support the recommendation that self-reported orofacial symptoms should be included in examination of children with JIA^28^.

The frequency of children with TMJ deformities was 38.8% at baseline which is consistent with previous studies^3–5^. Factors predicting TMJ deformities at baseline were number of years with disease and a smaller range of mandibular motion, i.e., MUO without pain. There was a small increase in the frequency of TMJ deformities at the two-year follow-up examination. However, this increase was smaller than described in older materials^29^.

CBCT findings on improvement or deterioration in TMJ morphology did not correlate with clinical findings. A possible explanation for these results may be the small number of joints that showed change in grade of deformity over time. Another explanation could be the timing of the examinations. The three “snapshots” of clinical variables in this study were not enough to detect flareups in disease activity. The fact that the number of joints showing deterioration was almost equal to the number of joints showing improvement, was an interesting observation. Previous studies have shown a pattern of progression in TMJ deformities over time^30^. This difference can probably be explained by the fact that the children in the current study to a large extent received highly effective medication (bDMARDS)^31^, implicating low disease activity. Further, there are other possible explanations for TMJ deformities in children than JIA involvement. TMJ injuries such as permanent disk displacement and trauma can lead to condylar deformity and growth disturbances, simulating changes due to JIA^32,33^. The same can be said for growth disturbances^33^. However, the prevalence of TMJ deformities confirmed on CBCT in healthy children is not known, so no comparison can be done.

Clinically relevant differences in mandibular range of motion were found between the children with TMJ deformities and those without. This insight may be a step towards setting cutoff values for MUO with and without pain for when to suspect JIA-related TMJ involvement. It is interesting and clinically relevant to note that there was a correlation between TMJ deformities and a larger discrepancy between MUO with and MUO without pain. However, the correlation was only seen at the two-year follow-up. This inconsistency, and the fact that it was seen at the two-year follow-up, may be explained by the children being familiar with the questions and the examination protocol at the two-year follow-up. In addition, the children were older and cognitively more mature in their pain assessment^34^.

In comparison to previous studies on healthy children, the children with JIA in the current study had a smaller range of mandibular motion. In age-matched children, the expected MUO ranges from 50 to 56 mm^35–37^. A lower MUO without pain at baseline was predictive of finding TMJ deformities on CBCT, whereas children without TMJ deformities exhibited MUO with pain equivalent to MUO for a normal population in the same age group. Previous studies have reported restricted mouth opening as the most frequently found clinical symptom in children with JIA^38,39^ with MUO < 35 mm in as many as 55% of the children in a study from 1982^38^. Here, we found that children with JIA have larger mandibular range of motion today compared to 40 years ago. The larger MUO, in this study, compared to earlier studies on children with JIA can probably be explained by improvements in pharmacological treatment. And the children were under regular supervision by pediatricians specialized in rheumatic disease.

In the current study, the OR for TMJ palpation pain was indicative of finding TMJ abnormalities on CBCT. Consensus-based recommendations for clinical examinations of TMJ status in children with JIA state that TMJ palpation pain has an intermediate predictive value for TMJ arthritis^20^ but should still be included in the clinical examination^20,28^. Our result confirms that palpation of TMJs (mouth closed) should be included in routine examinations of children with JIA.

Joint noises can be indicative of joint disease. Here, we report an association between crepitations and TMJ deformities found on CBCT, although a ROC-curve analyses showed poor level of discrimination according to Hosmer et al.^40^. The prevalence of TMJ noises found was high compared to expected prevalence numbers for the same age group^41^. For TMJ clickings, there is a wide spread in reported prevalence numbers for this age group and our results did not stand out as abnormal^42^. However, crepitations were present in almost 15% of our children. Crepitations are normally rare in younger children, prevalence numbers ranging from 0.2 to 1.0%^42^. In adults, crepitations are explained by changes in TMJ morphology and associated with disease such as osteoarthritis of the TMJ^43^,^44–46^ and underlying morphological factors for crepitation are most likely the same in children as in adults. To our knowledge, there are no previous publications on children/adolescents that correlate or assess the diagnostic validity of crepitations to alterations in TMJ morphology on CBCT.

When assessing TMJ involvement in JIA, a differential diagnosis such as TMD should be considered. Myalgia in masticatory muscles is associated with reduced MUO in both adults^47^ and children^48^. In the current study, palpatory pain in masticatory muscles was not related to change in TMJ morphology over time and it was also not associated with or predictive of finding TMJ deformities on CBCT. However, half of the children showed signs of myalgia in the masseter muscle at all three examinations. This finding is equivalent to those of a recent Norwegian study that concluded that children with JIA showed symptoms and clinical signs of TMD twice as often as healthy children and that approximately half of the children with JIA suffered from TMD^27^.

In this study, Angle class II malocclusion was twice as common as the expected prevalence in Swedish children 7 to 13 years old^49,50^. Since the Angle Class II occlusion did not correlate to TMJ deformities in this cohort, it might be due to an overall growth impairment which is common in chronic inflammatory conditions such as JIA^51^.

A strength of this study is that the participants are representative for the Swedish population of children with JIA in terms of distribution in sex, subtype of diagnosis, and pharmacological treatment^2^. In clinical research, there are limitations in participants eligible for inclusion as well as a timeframe to consider. As the sample size was relatively small, no analyses were made based on subtype of JIA. More girls than boys were included in the current study, this is consistent with the prevalence of the disease and a strength. However, for MUO, there is also a relationship to height; since boys usually are taller, they also have larger mandibular range of motion than girls^52^. Taken together, the number of girls may have affected our results toward smaller numbers for MUO.

The time frame of the study is a limitation. JIA is a chronic disease with a clinically remitting-relapsing pattern although believed to have an underlying continuous disease activity. Two years is a short time when looking at change in TMJ morphology and the design with three examinations one year apart cannot register every disease flareup or all changes in symptoms. The fact that there was a significant decrease in number of painful joints and a significant increase in children treated with bDMARDs over the study period indicate a high level of disease control in this cohort of children. This most definitely would have influenced the fact that there were few changes in TMJ deformities but could also be indicative for the status of children with JIA of today.

The lack of clinically validated and age-appropriate examination protocols for joint disease is a limitation and the underlying reason for this study. The RDC/TMD examination protocol was chosen because it was the only validated protocol available at study start. The drawback of this protocol is that it was originally developed for TMD evaluation of otherwise healthy adults; therefore, the study cannot account for factors such as growth or joint disease^43^. Nevertheless, the RDC/TMD has been used in several studies on adolescents^53–55^ as well as in numerous studies on adults with rhematic diseases^56^.

Taken together, this study offers the following recommendations for evaluation of TMJ involvement in JIA: Conduct regular and repeated clinical examinations focusing on change over time. For patient history, use targeted questions on TMJ pain and/or dysfunction at rest and during function (i.e., MUO and chewing). The clinical examination should encompass MUO without pain, MUO with pain, auscultation for TMJ noises (crepitations specifically), palpation of TMJs and masticatory muscles, and an assessment of occlusion/malocclusion/sagittal and vertical relations. Radiological examinations and imaging such as panoramic imaging CBCT, or MRI should be performed on individual indication.

In conclusion, in children with JIA self-reported TMJ, pain and dysfunction were common and predictive of TMJ deformities at baseline. In children with TMJ deformities, mandibular range of motion was smaller and palpatory TMJ pain as well as crepitations were more common. Nonetheless, most clinical variables were stable and showed only subtle variations from what is considered normal, indicating that although the TMJ is affected by JIA, the consequences are less severe in the post-biological era. However, the frequency of self-reported TMJ pain was high, and the TMJ was the third most common self-reported painful joint, at both baseline and the follow-up examinations. This underlines the importance of providing children adequate management of orofacial complications of JIA.

## Material and methods

A prospective longitudinal cohort study was initiated in 2011 at the Department of Orofacial Pain and Jaw Function at Eastmaninstitutet, Folktandvården Stockholm AB. The study consisted of three repeated clinical examinations that were conducted at baseline and after 12 and 24 months. At baseline and at the final two-year examination, CBCTs were performed.

The study was approved by the regional ethics committee in Stockholm (Dnr: 2010/2089-31/2). The study was also approved by the local radiation protection committee at Folktandvården Stockholm AB, Sweden. The study was registered at ClinicalTrials.gov (Protocol id: 2010/2089-31/2) and performed in accordance with the Declaration of Helsinki. Written and verbal information was given and written parental consent were obtained before the start of the study.

### Recruitment

Between November 2011 and June 2015, a group of children, 7–14 years of age, were enrolled. All the children were diagnosed with JIA according to the International League of Associations for Rheumatology (ILAR). The children were referred from pediatric rheumatologists at Astrid Lindgren Children’s Hospital at the Karolinska University Hospital, Solna, Sweden or Sachsska’s Children and the Youth Hospital at Södersjukhuset, Stockholm. In accordance with the Swedish odontological health program, all children receiving a diagnosis of JIA (in the Stockholm area) are referred to Eastmaninstitutet, where they are regularly examined for signs of TMJ arthritis and TMJ abnormalities.

Level of JIA disease activity for the included children were difficult to determine. However, only children that were under regular medical supervision and treatment were included whereas children perceived to be in long-time remission without pharmacological treatment did not meet inclusion criteria. Exclusion criteria were a concomitant diagnosis of any other autoimmune or autoinflammatory disease.

### Participants

**Figure 2:**
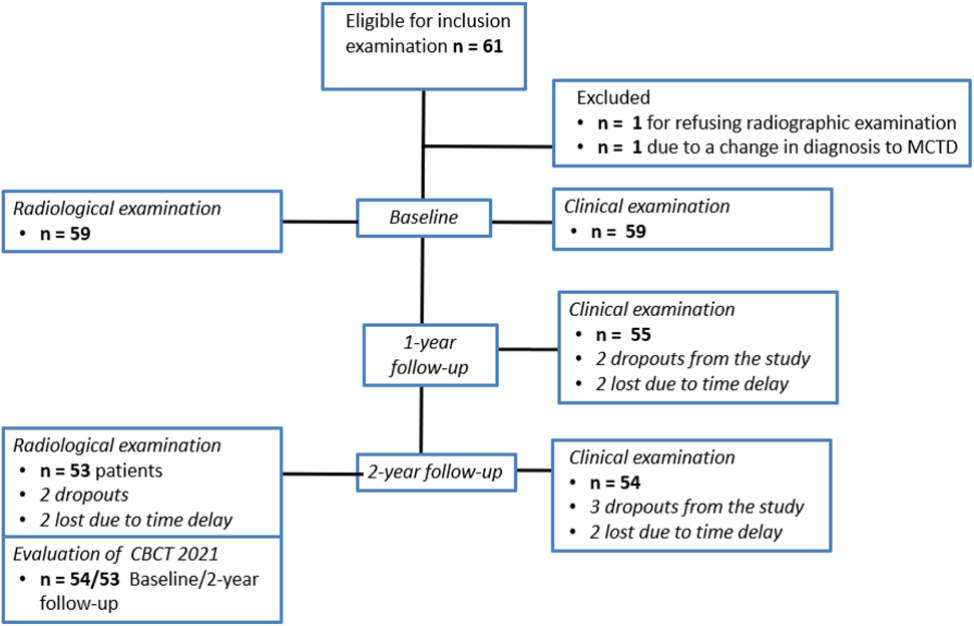
CONSORT Flowchart of participant enrollment and retainment.

At baseline, 61 children were enrolled in the study. Two children were excluded, one due to a change in diagnosis (from JIA to mixed connective tissue disease) and one for not participating in baseline radiographic examinations. In this study, data for 54 children who participated in all examinations are reported. For more information about recruitment and retention of participants, see Fig. 2.

Information on medical history, specifics on diagnosis, general disease activity, and medication was obtained from medical records by the pediatric rheumatologists. Baseline demographic data and participant characteristics for the 54 patients were recently described^57^.

### Study protocol

The clinical examinations were conducted according to the Research Diagnostic Criteria for TMD (RDC/TMD)^42^. This examination protocol includes assessment of mandibular range of motion, pain upon jaw movement, presence of TMJ sounds, as well as palpatory pain of the TMJ and jaw muscles. A couple of deviations were made from the RDC/TMD clinical examination protocol, and only Axis I was used. Maximum assisted mouth opening was not included and maximum unassisted mouth opening (MUO) with and without pain was defined as the vertical distance in millimeters between the incisal edges of the maxillary and mandibular central incisors plus the vertical overbite. In addition to the RDC/TMD protocol, data on occlusion and relation of malocclusion according to Angle^58^ were recorded. Furthermore, data on medical history, self-reported pain assessed with a 0–10 numeric rating scale (NRS)^59^, and self-reported functional disturbances were collected. We used pain drawings, visual aids, and specific questions on pain localization in resting position, in maximal mouth opening, and during clenching to record perceived localized pain. Examinations were performed by three investigators (specialists in Orofacial pain).

### Radiological examinations

Radiological examinations took place within a week after clinical examinations at baseline and at the two-year follow-up. CBCT examinations were performed at the Department of Oral and Maxillofacial Radiology at Eastmaninstitutet, Folktandvården Stockholm AB. CBCT examinations were performed with NewTom 3G (QR, Verona, Italy) (settings: 110 kV, 5–6 mA, 5.4 s), ProMax 3D Classic (Planmeca Oy, Helsinki, Finland) (settings: 90 kV, 9–10 mA, 12.3 s), and the most recent CBCTs in the study were taken with a 3D Accuitomo 170 (Morita, Kyoto, Japan) (settings: 85 kV, 7 mA, 17.5 s).

During 2021, the images were evaluated by three specialists in oral and maxillofacial radiology. Due to technical complications during transferal of DICOM files, CBCTs from 54 individuals were evaluated (54 CBCTs from baseline and 53 CBCTs from two-year follow-up) (Fig. 1). TMJ deformity was assessed using a grading system^60^, which is a modification of the grading system developed by Arvidsson et al.^30^. The classification for osseous TMJ deformities take in to account bone structures, shape and size of condyle and fossa/eminence In addition to the grading of TMJ deformity findings such as erosions, condylar sclerosis and condylar osteophytes were also recorded^60^. Any discrepancies between the three observers in the grading or in the assessment of additional findings were resolved by consensus discussions.

### Statistics

The statistical analyses were performed with the Statistical Package for the Social Sciences (IBM SPSS Statistics for Windows, Version 28.0; IBM, NY, USA). Descriptive data are presented as mean ± SD or median and interquartile range (IQR) if the data were skewed. The significance level was primarily set at *p* < 0.05 and at *p* < 0.0167 for post hoc testing. Data were processed and analyzed for the whole group as well as divided by presence of TMJ deformity seen on CBCT.

Differences between examinations in the study were tested with parametric or non-parametric tests depending on whether data showed normal distribution. For categorical variables or variables not normally distributed, Mann–Whitney U-test was applied to study differences between groups. For ordinal variables (more than two categories) and change, the Marginal Homogeneity test was used. The measure of the strength of association was tested with Goodman and Kruskal’s λ, Fischer’s exact test, Kendall’s tau-b (τb), and Odds Ratio.

For repeated measurements, Friedman test for nonparametric continuous variables was used and Bonferroni was used as post hoc test when the Friedman test showed significance. Exact Cochran’s Q was used to determine differences in dichotomous variables over time and for significant results post hoc analyses were made with multiple McNemar’s exact with Bonferroni correction.

Binominal logistic regression was performed to predict a dichotomous dependent (TMJ deformity on CBCT) variable given one or more independent variables. The ability of the binomial logistic regression model to discriminate individuals with and without the event of interest was tested with a ROC curve analysis.

## Data Availability

The data that support the findings of this study are available from the corresponding author, MC, upon reasonable request.
